# Supplementary material for: CSF proteome profiling reveals biomarkers to discriminate dementia with Lewy bodies from Alzheimer´s disease
Source: Nat Commun. 2023 Sep 13;14:5635. doi: 10.1038/s41467-023-41122-y (PMC10499811; doi:10.1038/s41467-023-41122-y)
Supplement: Supplementary file 1 — Supplementary Information [file 41467_2023_41122_MOESM1_ESM.pdf]

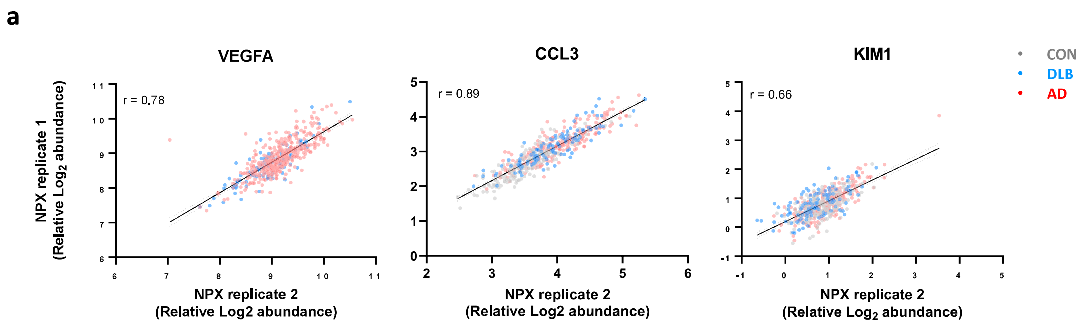

Supplementary figure 1. Correlations between protein replicates measured through different PEA panels. a, Scatter plots show the correlation of the NPX values (protein abundance) between the protein replicates that showed significant differences across the groups of interest in the complete cohort (n=534). Regression line and 95% confident intervals are depicted. Insert indicate the spearman correlation coefficient. DLB, Dementia with Lewy Bodies; CON, cognitively unimpaired controls; AD, Alzheimer's disease

Supplementary figure 2

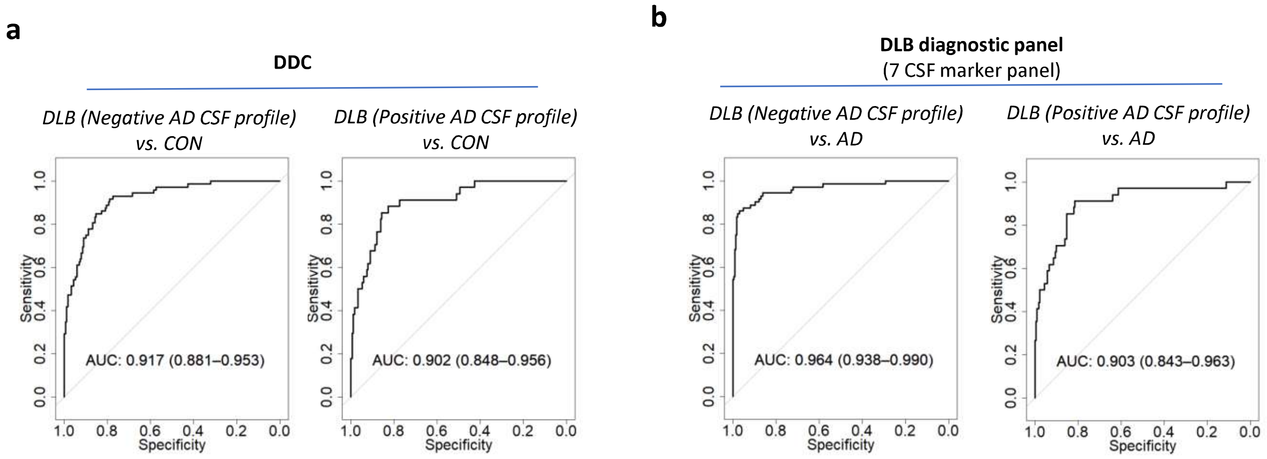

Supplementary figure 2. Additional sensitivity analysis for the CSF biomarker panels discriminating DLB from controls or AD a) Receiver operating characteristic (ROC) curves showed that DDC (a) or the CSF DLB-diagnostic panel (b) could discriminate DLB from controls or AD with similar performance independent of the presence of AD co-pathology in DLB patients (as measured by positive or negative AD CSF biomarker profiles). Inserts outline corresponding AUC and 95% CI. DLB, dementia with Lewy bodies; CON, cognitively unimpaired controls; AD, Alzheimer's disease;

Supplementary figure 3

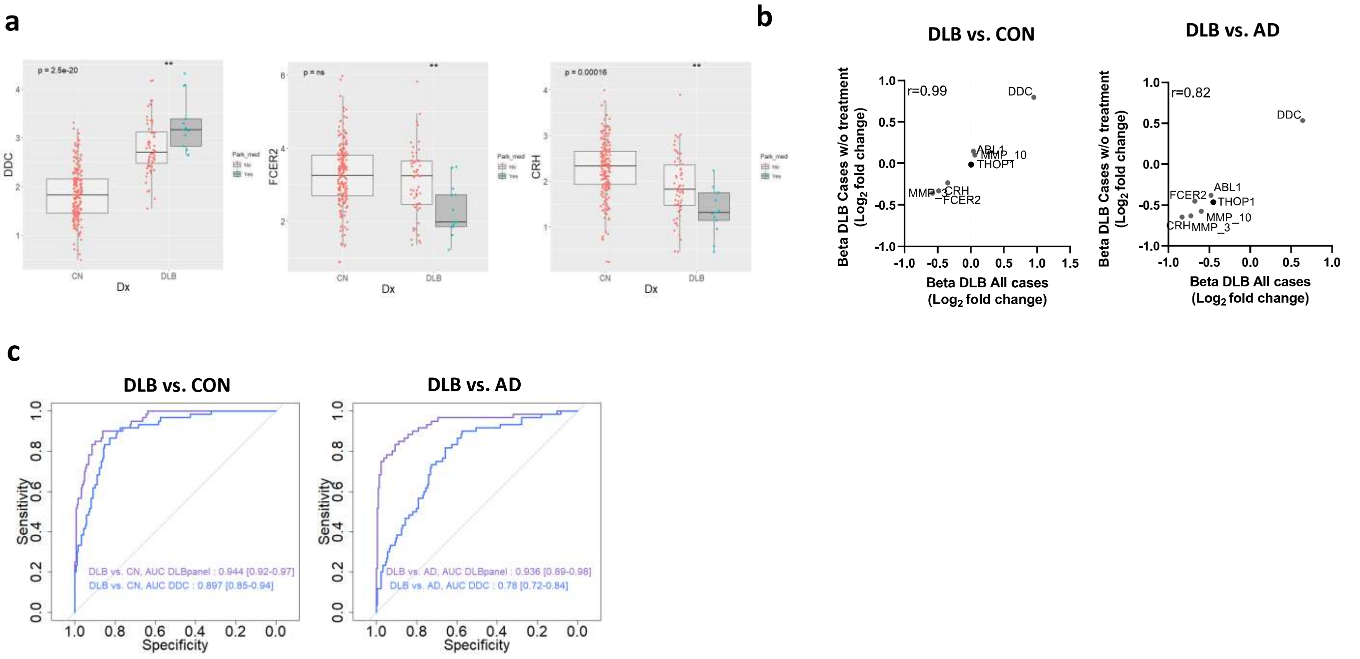

Supplementary figure 3. Sensitivity analysis of parkinsonian related medication on the CSF proteins within the biomarker panel. a) Box plots represent the abundance (log2 NPX) of CSF DDC, FCER2 and CRH in Controls (n=190) and DLB cases stratified by parkinsonian medication status (60 with no medication in red and 13 with medication in blue) at time of samples collection. Asterisks represent significance in CSF protein levels within the DLB group based on medication status. Insert indicate p value of the differential protein abundance between controls and DLB with no medication after ANOVA analysis. **b**) Scatter plots show the correlation between the beta-coefficients obtained in the complete discovery cohorts to those obtained when only patients that did not have parkinsonian related medication were included in the analysis (DLB=60, CON=190, AD=216). Insert indicate the spearman correlation coefficient**. c)**Receiver operating characteristic (ROC) curves depicting the performance of (b) DDC or (c) the CSF DLB-diagnostic panel in the comparison between DLB (n=60) and controls (n=190) or AD (n=217) in the discovery cohorts after including patients that are not undergoing any parkinsonian related treatment. Inserts outline corresponding AUC and 95% CI. DLB, dementia with Lewy bodies; CON, cognitively unimpaired controls; AD, Alzheimer's disease.

Supplementary figure 4

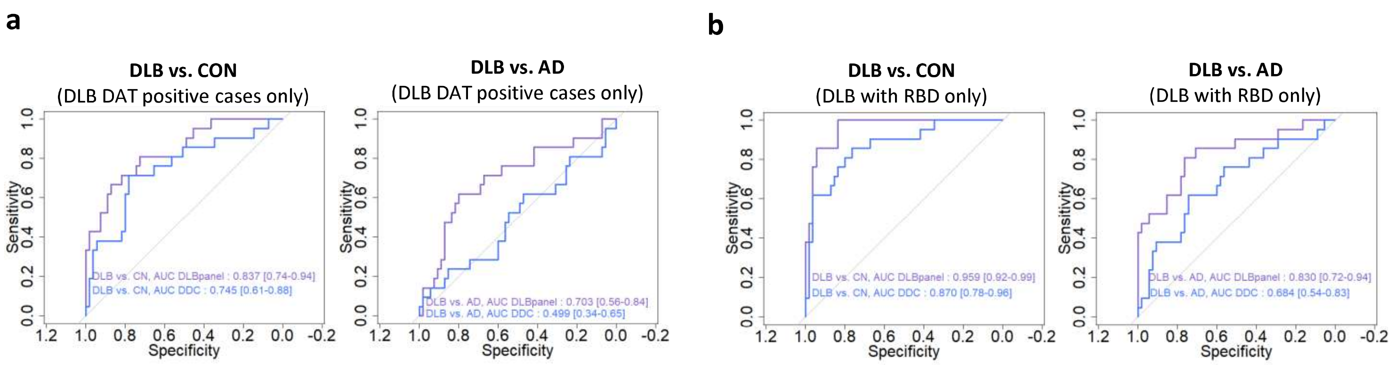

Supplementary figure 4. Sensitivity analysis of the presence of abnormal DAT scan or RBD positivity on the performance of the CSF biomarker panel to discriminate DLB from controls or AD in the second validation cohort. a) Receiver operating characteristic (ROC) curves depicting the performance of DDC (blue) or the CSF DLB-diagnostic panel (purple) in the comparison between DLB (n=21) and controls (n=55) or AD (n=55) in the second validation cohort after including only DLB cases with abnormal DaTScan. b) ROCs depicting the performance of DDC (blue) or the CSF DLB-diagnostic panel (purple) in the comparison between DLB (n=21) and controls (n=55) or AD (n=55) in the second validation cohort after including only DLB cases with RBD. Inserts outline corresponding AUC and 95% CI. DLB, dementia with Lewy bodies; CON, cognitively unimpaired controls; AD, Alzheimer's disease; DAT, FPCIT single-photon emission computed tomography; RBD, REM Sleep Behavior Disorder.

Supplementary figure 5

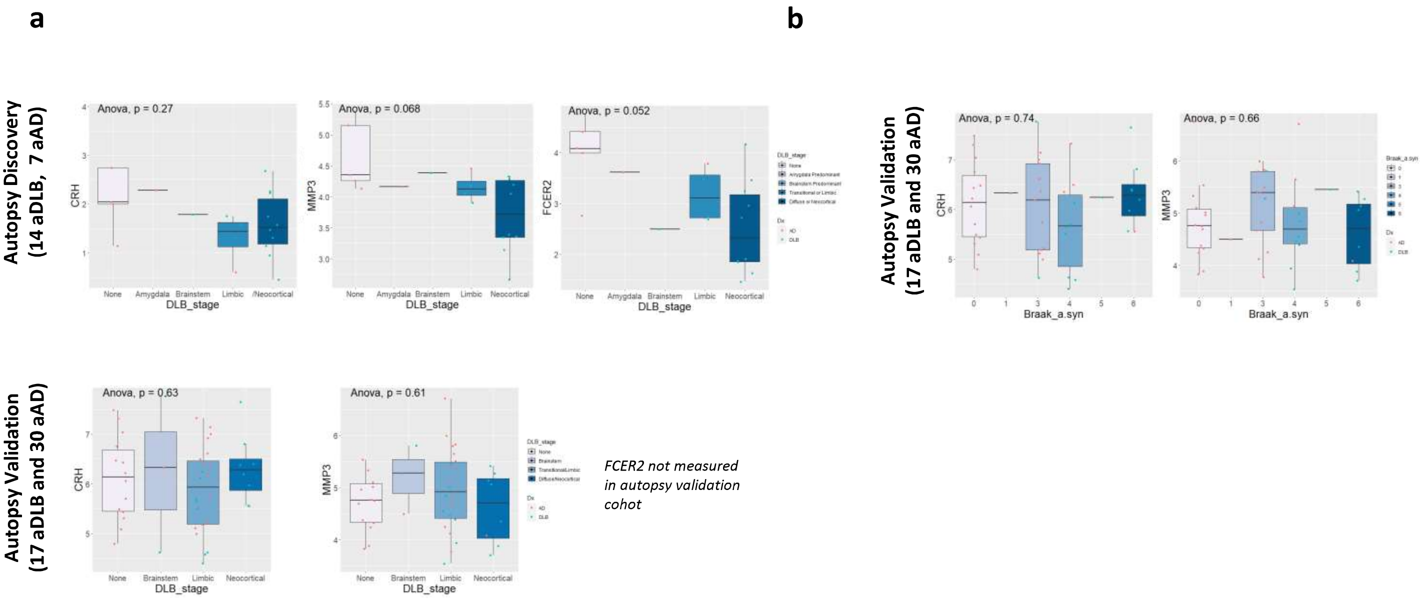

Supplementary figure 5. Associations of CSF FCER2, CRH and MMP3 with DLB and a-syn Braak stage in the autopsy confirmed cases from the discovery and autopsy validation cohort. a) Box plot represent the abundance (log2 NPX) of the different CSF proteins across DLB stages in a subset of cases from the discovery cohort that were autopsy confirmed (Autopsy discovery, 14 aDLB and 7 aAD) and the autopsy confirmed validation cohort (17 aDLB and 30 aAD). b) Box plot represent the abundance (log2 NPX) of the different CSF proteins across a-syn Braak stages in the autopsy confirmed validation cohort (a-syn Braak stage data was not available from the autopsy confirmed cases of the Discovery cohort). Difference across groups were analyzed by Anova. Inserts depict the corresponding p value. aDLB, autopsy confirmed dementia with Lewy bodies; aAD, autopsy confirmed Alzheimer's disease.

Supplementary Table 1. Quality parameters of custom PEA assays

Protein name	Assay	Uniport	LOD	CSF missingness (%)	Intra-CV (%)	Inter-CV (%)
Aromatic-L-amino-acid decarboxylase	DDC	P20711	1.98	1%	5%	8%
Corticoliberin	CRH	P06850	1.60	1%	7%	18%
Matrix metalloproteinase-3	MMP-3	P08254	0.38	1%	4%	8%
Tyrosine-protein kinase ABL1	ABL1*	P00519	1.01	10%	5%	2%
Matrix metalloproteinase-10	MMP-10	P09238	0.98	1%	5%	8%
Thimet oligopeptidase	THOP1	P52888	1.38	1%	5%	8%
			Mean	3%	5%	9%

CSF missingness is the % of samples whose values were < the corresponding LOD. CVs caculated using quality control samples. *For ABL1 CVs were calculated using assay calibrators, as QC samples reported values <LOD. LOD, Lower limit of detection. CV, coefficient of variation.

Supplementary Table 2. Demographic characteristics PD cohorts

	AMP-PD cohort			(LONI)-PPMI cohort	
	**CON (n=93)**	**proPD (n=44)**	**PD (n=33)**	**CON (n=37)**	**PD (n=36)**
**Age, years (Mean, SD)**	61 (10)[a,b]	68(6)[b,c]	63 6)[a,c]	60 (12)	61(12)
**Sex (M, %)**	64(69%)	27 (61%)	23 (70%)	27(73%)	26 (72%)
**UPDRS-III (total)[1]**	1(2)	4.6(5)	22(9)	1(2)	20(9)
**HNY stage (0,1,2;%)[2]**	98,2,0	93,5,2	0,45,55	36,1,0	0,44,56

Data are median (interquartile range) unless otherwise specified.

[1]Total UPDRS-III was used as a mesure of parkinsonism was available for every participant

[2]Hoen and Yahr Stage as a measure of functional disabilities was available for every participant

CON, controls; proPD, Prodromal PD; PD, Parkinson´s disease; SD, Standard desviation;M, Male.

Supplementary Table 3. Biomarkers supporting DLB diagnosis across cohorts

	Discovery cohort	Validation cohort 1	Validation cohort 2	Autopsy cohort
**Autopsy confirmed, n (%)**	14 (13%)	0 (0%)	0 (0%)	17 (100%)
**Clinical based diagnosis, n (%)**	95 (87%)	54 (100%)	55 (100%)	na
**DAT-SPECT, n abnormal (%)**	23 (24%)	19 (35%)	21 (38%)	na
**EEG, n abnormal (%)**	41 (43%)	28 (51%)	na	na
**No autopsy or supporting biomarkers available, n (%)**	48 (44%)	18 (33%)	27 (49%)	na

Abbreviations: DAT-SPECT: FPCIT single-photon emission computed tomography; EEG: electroencephalography.

Supplementary Table 4. Proteins that passed the quality control and were included in the analysis

Name	Abbreviaton	Uniprot	Olink panel(s)	CSF Detectavility
C-C motif chemokine 24	CCL24	O00175	CVD III	91.22%
Galectin-9	Gal-9	O00182	CVD II	100.00%
Galectin-8	gal-8	O00214	Neuro	99.90%
Tumor necrosis factor receptor superfamily member 10A	TNFRSF10A	O00220	CVD II, Cell-Reg	100%; 100%
Signal-regulatory protein beta-1	SIRPB1	O00241	Dev	100.00%
Agouti-related protein	AGRP	O00253	CVD II	100.00%
DNA fragmentation factor subunit alpha	DFFA	O00273	IR	100.00%
Osteoprotegerin	OPG	O00300	Inf, CVD III	100%; 100%
Matrilin-2	MATN2	O00339	Dev	100.00%
GDNF family receptor alpha-2	GFRA2	O00451	Cell-Reg	100.00%
Neural cell adhesion molecule L1-like protein	CHL1	O00533	C-Met	100.00%
Delta-like protein 1	DLL1	O00548	Onc II	100.00%
C-C motif chemokine 21	CCL21	O00585	Dev	96.79%
Podocalyxin	PODXL	O00592	Onc II	99.69%
Protein CYR61	CYR61	O00622	Onc II	100.00%
Neurocan core protein	NCAN	O14594	Neuro	100.00%
C-X-C motif chemokine 11	CXCL11	O14625	Inf	99.90%
TNF-related apoptosis-inducing ligand receptor 2	TRAIL-R2	O14763	CVD II	100.00%
Tripeptidyl-peptidase 1	TPP1	O14773	Dev	100.00%
Growth/differentiation factor 8	GDF-8	O14793	Neuro	100.00%
Tumor necrosis factor receptor superfamily member 10C	TNFRSF10C	O14798	CVD III	100.00%
Secretory carrier-associated membrane protein 3	SCAMP3	O14828	Onc II	100.00%
Tumor necrosis factor receptor superfamily member 13B	TNFRSF13B	O14836	CVD II	100.00%
Protein Wnt-9a	WNT9A	O14904	Cell-Reg	90.49%
Protocadherin-17	PCDH17	O14917	Cell-Reg	100.00%
Plexin-B2	PLXNB2	O15031	C-Met	100.00%
Angiopoietin-2	ANGPT2	O15123	Met	100.00%
Ephrin type-B receptor 6	EPHB6	O15197	Neuro	99.90%
Matrilin-3	MATN3	O15232	Neuro	100.00%
C-C motif chemokine 25	CCL25	O15444	Inf	97.52%
Toll-like receptor 3	TLR3	O15455	Onc II	100.00%
C-C motif chemokine 16	CCL16	O15467	CVD III	100.00%
Leucine-rich repeat transmembrane protein FLRT2	FLRT2	O43155	Neuro	99.90%
Plexin-B1	PLXNB1	O43157	Neuro	100.00%
Kallikrein-10	KLK10	O43240	Met	100.00%
Kunitz-type protease inhibitor 1	SPINT1	O43278	Dev	100.00%
Kunitz-type protease inhibitor 2	SPINT2	O43291	Dev	100.00%
Cochlin	COCH	O43405	Dev	100.00%
Beta-1,4-glucuronyltransferase 1	B4GAT1	O43505	Dev	100.00%
Tumor necrosis factor (Ligand) superfamily, member 12	TWEAK	O43508	Inf	100.00%
Bcl-2-like protein 11 isoform BimL	BCL2L11	O43521-2	Cell-Reg	100.00%
Tumor necrosis factor ligand superfamily member 14	TNFSF14	O43557	Inf	96.59%
Carbonic anhydrase 12	CA12	O43570	OD	99.48%
Protein sprouty homolog 2	SPRY2	O43597	IR	100.00%
Integral membrane protein 2A	ITM2A	O43736	IR	100.00%
Angiopoietin-related protein 7	ANGPTL7	O43827	Met	100.00%
EGF-like repeat and discoidin I-like domain-containing protein 3	EDIL3	O43854	OD	99.90%
Xaa-Pro aminopeptidase 2	XPNPEP2	O43895	Onc II	97.52%
Vascular endothelial growth factor D	VEGFD	O43915	CVD II, Cell-Reg	100%; 99,79%
C-X-C motif chemokine 13	CXCL13	O43927	Onc II	100.00%
Heparan-sulfate 6-O-sulfotransferase 1	HS6ST1	O60243	Cell-Reg	100.00%
Kallikrein-8	hK8	O60259	Onc II	100.00%
Neuropilin-2	NRP2	O60462	Neuro	99.69%
GDNF family receptor alpha-3	GDNFR-alpha-3	O60609	Neuro	100.00%
Cathepsin L2	CTSV	O60911	Onc II	100.00%
Low affinity immunoglobulin gamma Fc region receptor III-B	FCGR3B	O75015	C-Met	100.00%
Leukocyte immunoglobulin-like receptor subfamily B member 5	LILRB5	O75023	C-Met	99.28%
Immunoglobulin superfamily member 3	IGSF3	O75054	Cell-Reg	100.00%
Disintegrin and metalloproteinase domain-containing protein 23	ADAM 23	O75077	Neuro	100.00%
ICOS ligand	ICOSLG	O75144	Onc II	100.00%
Semaphorin-7A	SEMA7A	O75326	Dev	100.00%
Ectonucleoside triphosphate diphosphohydrolase 6	ENTPD6	O75354	OD, Cell-Reg	100%; 99,79%
Ectonucleoside triphosphate diphosphohydrolase 5	ENTPD5	O75356	Met	98.97%
Tumor necrosis factor receptor superfamily member 21	TNFRSF21	O75509	Neuro	100.00%
Peptidoglycan recognition protein 1	PGLYRP1	O75594	CVD III	100.00%
Tumor necrosis factor ligand superfamily member 13	TNFSF13	O75888	Onc II	100.00%
N(G),N(G)-dimethylarginine dimethylaminohydrolase 1 (DDAH1)	DDAH1	O94760	Cell-Reg	100.00%
Contactin-5	CNTN5	O94779	Neuro	100.00%
Dickkopf-related protein 1	Dkk-1	O94907	CVD II	100.00%
Vesicle-associated membrane protein 5	VAMP5	O95183	Cell-Reg	89.45%
Netrin receptor UNC5C	UNC5C	O95185	Neuro	99.90%
WNT1-inducible-signaling pathway protein 1	WISP-1	O95388	Onc II	100.00%
Tumor necrosis factor receptor superfamily member 6B	TNFRSF6B	O95407	Onc II	100.00%
Apolipoprotein M	APOM	O95445	C-Met	98.76%
Neuronal pentraxin receptor	NPTXR	O95502	Met	100.00%
NAD kinase	DK	O95544	Met	99.90%
Follistatin-related protein 3	FSTL3	O95633	Dev	100.00%
Synaptosomal-associated protein 29	SP29	O95721	Dev	100.00%
Cytotoxic and regulatory T-cell molecule	CRTAM	O95727	Neuro	92.98%
Fibroblast growth factor 19	FGF-19	O95750	Inf	100.00%
Angiopoietin-related protein 1	ANGPTL1	O95841	Met	100.00%
CD160 antigen	CD160	O95971	Onc II	99.90%

Interleukin-18-binding protein	IL-18BP	O95998	CVD III	100.00%
Superoxide dismutase (Cu-Zn)	SOD1	P00441	C-Met	100.00%
Tyrosine-protein kinase ABL1	ABL1	P00519	Onc II	100.00%
Epidermal growth factor receptor	EGFR	P00533	CVD III	100.00%
Urokinase-type plasminogen activator	uPA	P00749	Inf, CVD III	100%; 100%
Tissue-type plasminogen activator	t-PA	P00750	CVD III	100.00%
Adenosine Deaminase	ADA	P00813	Inf	100.00%
Carbonic anhydrase 2	CA2	P00918	Dev	91.11%
Serine protease inhibitor Kazal-type 1	SPINK1	P00995	Dev	100.00%
Metalloproteinase inhibitor 1	TIMP1	P01033	C-Met	100.00%
Cystatin-C	CST3	P01034	C-Met	100.00%
Platelet-derived growth factor subunit B	PDGF subunit B	P01127	CVD II	100.00%
Low-density lipoprotein receptor	LDL receptor	P01130	CVD III	100.00%
Transforming growth factor alpha	TGF-alpha	P01135	Inf, Onc II	100%; 100%
Latency-associated peptide transforming growth factor beta-1	LAP TGF-beta-1	P01137	Inf	100.00%
Beta-nerve growth factor	Beta-NGF	P01138	Inf, Neuro	100%; 99,9%
Glycoprotein hormones alpha chain	CGA	P01215	Dev	100.00%
Growth hormone	GH	P01241	CVD II	98.76%
Calcitonin	CALCA	P01258	OD	99.90%
Pancreatic prohormone	PPY	P01298	Onc II	100.00%
TNF-beta	TNFB	P01374	Inf	98.24%
Interleukin-2 receptor subunit alpha	IL2-RA	P01589	CVD III	99.48%
T-cell surface glycoprotein CD4	CD4	P01730	CVD II	100.00%
Myoglobin	MB	P02144	CVD III	100.00%
Collagen alpha-1(I) chain	COL1A1	P02452	CVD III	100.00%
Collagen alpha-1(IV) chain	COL4A1	P02462	Cell-Reg	100.00%
Protein AMBP	AMBP	P02760	CVD II	100.00%
C-X-C motif chemokine 10	CXCL10	P02778	Inf	100.00%
Transferrin receptor protein 1	TR	P02786	CVD III	99.79%
Angiogenin	ANG	P03950	C-Met	100.00%
Coagulation factor XI	F11	P03951	C-Met	100.00%
Matrix metalloproteinase-1	MMP-1	P03956	Inf	100.00%
Tissue alpha-L-fucosidase	FUCA1	P04066	Dev	100.00%
Vitamin K-dependent protein C	PROC	P04070	C-Met	100.00%
Cystatin-B	CSTB	P04080	CVD III	100.00%
Platelet-derived growth factor subunit A	PDGF subunit A	P04085	CVD III	100.00%
Superoxide dismutase (Mn), mitochondrial	SOD2	P04179	CVD II	100.00%
Thy-1 membrane glycoprotein	THY 1	P04216	Neuro	100.00%
HLA class II histocompatibility antigen gamma chain	CD74	P04233	Dev	99.38%
von Willebrand factor	vWF	P04275	CVD III	97.52%
Receptor tyrosine-protein kinase erbB-2	ErbB2/HER2	P04626	Onc II	100.00%
Heat shock 27 kDa protein	HSP 27	P04792	CVD II	100.00%
Amyloid beta A4 protein	APP	P05067	Dev	100.00%
Integrin beta-2	ITGB2	P05107	CVD III	100.00%
Plasminogen activator inhibitor 1	PAI	P05121	CVD III	100.00%
Plasma serine protease inhibitor	SERPINA5	P05154	C-Met	100.00%
Interleukin-6	IL6	P05231	Inf, Onc II, CVD II, IR	100%; 100%; 100%; 100%
Intercellular adhesion molecule 1	ICAM1	P05362	C-Met	100.00%
Lithostathine-1-alpha	REG1A	P05451	C-Met	100.00%
Thyroxine-binding globulin	SERPINA7	P05543	C-Met	99.59%
Integrin beta-1	ITGB1	P05556	Dev	100.00%
T-cell surface glycoprotein CD5	CD5	P06127	Inf	100.00%
Complement C2	C2	P06681	C-Met	100.00%
Low affinity immunoglobulin epsilon Fc receptor	FCER2	P06734	Dev	100.00%
Integrin alpha-V	ITGAV	P06756	Onc II	100.00%
Corticoliberin	CRH	P06850	OD	99.59%
Lipoprotein lipase	LPL	P06858	CVD II	99.90%
Thrombomodulin TM		P07204	CVD II	100.00%
Protein disulfide-isomerase	P4HB	P07237	Dev	95.14%
Cathepsin D	CTSD	P07339	CVD III	100.00%
Platelet glycoprotein Ib alpha chain	GP1BA	P07359	C-Met	90.70%
Carbonic anhydrase 3	CA3	P07451	C-Met	92.77%
Trypsin-2	PRSS2	P07478	C-Met	99.59%
Decorin	DCN	P07585	CVD II	100.00%
Cathepsin L1	CTSL1	P07711	CVD II	100.00%
Tyrosine-protein kinase Yes	YES1	P07947	OD	99.79%
Tyrosine-protein kinase Lyn	LYN	P07948	Onc II	98.24%
Proto-oncogene tyrosine-protein kinase receptor Ret	RET	P07949	Onc II	100.00%
Insulin-like growth factor 1 receptor	IGF1R	P08069	Onc II	100.00%
Beta-glucuronidase	GUSB	P08236	Dev	100.00%
Matrix metalloproteinase-2	MMP-2	P08253	CVD III	100.00%
Matrix metalloproteinase-3	MMP-3	P08254	CVD III	100.00%
Neprilysin	NEP	P08473	Neuro	99.69%
Hepatocyte growth factor receptor	MET	P08581	C-Met	100.00%
Vimentin	VIM	P08670	Onc II	94.63%
Coagulation factor VII	F7	P08709	C-Met	88.43%
Insulin-like growth factor-binding protein 1	IGFBP-1	P08833	CVD III	100.00%
Interleukin-6 receptor subunit alpha	IL-6RA	P08887	CVD III	100.00%
Gamma-enolase	ENO2	P09104	Met	100.00%
Matrix metalloproteinase-7	MMP-7	P09237	CVD II	100.00%
Matrix metalloproteinase-10	MMP-10	P09238	Inf	99.79%
CD48 antigen	CD48	P09326	Onc II	100.00%
C-X-C motif chemokine 1	CXCL1	P09341	Inf,CVD II	100%; 100%
Galectin-1	Gal-1	P09382	Onc II	100.00%

Dihydropteridine reductase	QDPR	P09417	Met	100.00%
SPARC	SPARC	P09486	Onc II	100.00%
Heme oxygenase 1	HO-1	P09601	CVD II	99.90%
Macrophage colony-stimulating factor 1	CSF-1	P09603	Inf	100.00%
Platelet-derived growth factor receptor beta	PDGFRB	P09619	Dev	100.00%
Pro-cathepsin H	CTSH	P09668	Met	100.00%
Tumor-associated calcium signal transducer 2	TACSTD2	P09758	Cell-Reg	100.00%
Furin	FUR	P09958	Onc II	100.00%
Cryptic protein	CFC1	P0CG37	Cell-Reg	100.00%
Ig lambda-2 chain C regions	IGLC2	P0DOY2	C-Met	100.00%
Granzyme B	GZMB	P10144	Onc II	85.74%
Interleukin-8	IL-8	P10145	Inf	100.00%
C-C motif chemokine 3	CCL3	P10147	Inf, CVD II	100%; 100%
Osteopontin	OPN	P10451	CVD III	100.00%
Receptor-type tyrosine-protein phosphatase F	PTPRF	P10586	Dev	100.00%
Tissue factor pathway inhibitor	TFPI	P10646	CVD III	100.00%
Mast/stem cell growth factor receptor Kit	KIT	P10721	C-Met	100.00%
Mannose-binding protein C	MBL2	P11226	C-Met	99.48%
Cation-independent mannose-6-phosphate receptor	IGF2R	P11717	Dev	100.00%
Fatty acid-binding protein, intestinal (FABP2)	FABP2	P12104	CVD II	97.00%
Low affinity immunoglobulin gamma Fc region receptor II-a	FCGR2A	P12318	C-Met	99.38%
Granzyme A	GZMA	P12544	Neuro	99.90%
Bone morphogenetic protein 4	BMP-4	P12644	Neuro	99.17%
Eosinophil cationic protein	RSE3	P12724	Met	99.90%
Cadherin-1	CDH1	P12830	C-Met	100.00%
Interleukin-7	IL-7	P13232	Inf	97.93%
C-C motif chemokine 4	CCL4	P13236	Inf	100.00%
Monocyte chemotactic protein 1	MCP-1	P13500	Inf, CVD III	100%; 100%
Neural cell adhesion molecule 1	NCAM1	P13591	C-Met	100.00%
Intercellular adhesion molecule 2	ICAM-2	P13598	CVD III	100.00%
Versican core protein	VCAN	P13611	Met	100.00%
Tartrate-resistant acid phosphatase type 5	TR-AP	P13686	CVD III	100.00%
Carcinoembryonic antigen-related cell adhesion molecule 1	CEACAM1	P13688	Onc II	99.79%
Tissue factor	TF	P13726	CVD II	100.00%
CD59 glycoprotein	CD59	P13987	C-Met	100.00%
L-selectin	SELL	P14151	C-Met	100.00%
Macrophage migration inhibitory factor	MIF	P14174	Dev	100.00%
Hepatocyte growth factor	HGF	P14210	Inf, Onc II	100%; 100%
Carboxypeptidase M	CPM	P14384	Neuro	99.90%
Nidogen-1	NID1	P14543	C-Met	100.00%
Interleukin-1 receptor type 1	IL-1RT1	P14778	CVD III	100.00%
Carboxypeptidase A1	CPA1	P15085	CVD III	100.00%
Carboxypeptidase B	CPB1	P15086	CVD III	100.00%
Fatty acid-binding protein, adipocyte (FABP4)	FABP4	P15090	CVD III	100.00%
Aminopeptidase N	AP-N	P15144	CVD III	100.00%
Poliovirus receptor	PVR	P15151	Neuro	99.90%
Interferon gamma receptor 1	IFN-gamma-R1	P15260	Onc II	100.00%
Arylsulfatase A	ARSA	P15289	Dev	100.00%
Beta-1,4-galactosyltransferase 1	B4GALT1	P15291	Dev	100.00%
Ezrin	EZR	P15311	Neuro	100.00%
Folate receptor alpha	FR-alpha	P15328	Onc II	100.00%
Granulocyte-macrophage colony-stimulating factor receptor subunit alpha	GM-CSF-R-alpha	P15509	Neuro	100.00%
Amphiregulin	AREG	P15514	Onc II, IR	97.42%
Membrane cofactor protein	CD46	P15529	C-Met	100.00%
Vascular endothelial growth factor A	VEGF-A	P15692	Inf, Onc II	100%; 100%
Arylsulfatase B	ARSB	P15848	Cell-Reg	100.00%
Beta-galactoside alpha-2,6-sialyltransferase 1	ST6GAL1	P15907	C-Met	100.00%
P-selectin	SELP	P16109	CVD III	100.00%
Aggrecan core protein	ACAN	P16112	Dev	100.00%
Platelet-derived growth factor receptor alpha	PDGF-R-alpha	P16234	Neuro	100.00%
Beta-galactosidase	GLB1	P16278	IR	97.31%
Platelet endothelial cell adhesion molecule	PECAM-1	P16284	CVD III	100.00%
Epithelial cell adhesion molecule	Ep-CAM	P16422	CVD III	100.00%
Cysteine-rich secretory protein 2	CRISP2	P16562	Cell-Reg	100.00%
E-selectin	SELE	P16581	CVD III	100.00%
Carboxypeptidase E	CPE	P16870	Onc II	100.00%
Sphingomyelin phosphodiesterase	SMPD1	P17405	Neuro	99.90%
Endoglin	ENG	P17813	C-Met	95.45%
Galectin-3	Gal-3	P17931	CVD III	100.00%
Insulin-like growth factor-binding protein 3	IGFBP3	P17936	C-Met	100.00%
Insulin-like growth factor-binding protein 2	IGFBP-2	P18065	CVD III	100.00%
Integrin beta-5	ITGB5	P18084	Onc II	100.00%
Interleukin-1 receptor antagonist protein	IL-1ra	P18510	CVD II	100.00%
Syndecan-1	SYND1	P18827	Onc II	100.00%
Peptidyl-glycine alpha-amidating monooxygenase	PAM	P19021	C-Met	100.00%
Cadherin-2	CDH2	P19022	Met	100.00%
Lymphocyte function-associated antigen 3	CD58	P19256	Dev	100.00%
Vascular cell adhesion protein 1	VCAM1	P19320	C-Met	100.00%
Tumor necrosis factor receptor 1	TNF-R1	P19438	CVD III	100.00%
Follistatin	FS	P19883	CVD II	100.00%
Elafin	PI3	P19957	CVD III	98.35%
Thymidine phosphorylase	TYMP	P19971	Met	100.00%
Complement receptor type 2	CR2	P20023	C-Met	97.62%
Transcobalamin-2	TCN2	P20062	C-Met	100.00%

Tumor necrosis factor receptor 2	TNF-R2	P20333	CVD III	100.00%
Parvalbumin alpha	PVALB	P20472	OD	100.00%
Aromatic-L-amino-acid decarboxylase	DDC	P20711	Met	99.90%
Pleiotrophin	PTN	P21246	OD	100.00%
Stem cell factor	SCF	P21583	Inf, CVD II,Onc II	100%; 100%; 100%
5'-nucleotidase	5'-NT	P21589	Onc II	100.00%
Midkine	MK	P21741	Onc II	100.00%
Macrophage scavenger receptor types I and II	MSR1	P21757	Neuro	99.90%
Biglycan	BGN	P21810	Cell-Reg	90.49%
Receptor tyrosine-protein kinase erbB-3	ErbB3/HER3	P21860	Onc II	100.00%
Protein-glutamine gamma-glutamyltransferase 2	TGM2	P21980	CVD II	100.00%
Bone morphogenetic protein 6	BMP-6	P22004	CVD II	100.00%
Tenascin-X	TNXB	P22105	C-Met	94.01%
Cadherin-3	CDH3	P22223	Neuro	99.90%
Galanin peptides	GAL	P22466	Met	100.00%
Carbonic anhydrase 4	CA4	P22748	C-Met	100.00%
Peptidyl-prolyl cis-trans isomerase B	PPIB	P23284	Dev	100.00%
Oligodendrocyte-myelin glycoprotein	OMG	P23515	Cell-Reg	100.00%
C-type natriuretic peptide	NPPC	P23582	OD	99.90%
Corticotropin-releasing factor-binding protein	CRHBP	P24387	Dev	99.90%
Insulin-like growth factor-binding protein 6	IGFBP6	P24592	C-Met	100.00%
Tenascin	TNC	P24821	C-Met	94.32%
Proteinase-activated receptor 1	PAR-1	P25116	CVD II	99.79%
Tumor necrosis factor receptor superfamily member 6	FAS	P25445	CVD III	100.00%
Cathepsin S	CTSS	P25774	Neuro	100.00%
CD40L receptor	CD40	P25942	Inf	100.00%
Protein S100-A4	S100A4	P26447	Onc II	90.39%
CD27 antigen	CD27	P26842	Onc II	100.00%
Dipeptidyl peptidase 4	DPP4	P27487	C-Met	99.79%
DNA-(apurinic or apyrimidinic site) lyase	APEX1	P27695	Met	93.60%
Calreticulin	CALR	P27797	OD	85.74%
Interleukin-1 receptor type 2	IL-1RT2	P27930	CVD III	98.45%
Cystatin D	CST5	P28325	Inf	100.00%
Granulins	GRN	P28799	CVD III	100.00%
ADP-ribosyl cyclase/cyclic ADP-ribose hydrolase 1	CD38	P28907	Neuro	100.00%
T-cell surface glycoprotein CD1c	CD1C	P29017	Met	99.48%
Ephrin type-A receptor 2	EPHA2	P29317	Onc II	100.00%
Interleukin-12 subunit beta	IL-12B	P29460	Inf	100.00%
Interleukin-12	IL-12	P29460,P29459	Neuro	99.90%
Peroxiredoxin-5	PRDX5	P30044	IR	85.63%
Phosphatidylethanolamine-binding protein 1	PEBP1	P30086	Dev	100.00%
Tyrosine-protein kinase receptor UFO	AXL	P30530	CVD III	100.00%
Alpha-2-macroglobulin receptor-associated protein	Alpha-2-MRAP	P30533	Neuro	99.90%
Syndecan-4	SDC4	P31431	Met	100.00%
Protein S100-A11	S100A11	P31949	Onc II	96.90%
Glypican-1	GPC1	P35052	Onc II	100.00%
Serpin B6	SERPINB6	P35237	Met	100.00%
ADM	ADM	P35318	CVD II	100.00%
Thrombospondin-2	THBS2	P35442	CVD II	100.00%
Thrombospondin-4	THBS4	P35443	C-Met	100.00%
Alpha-L-iduronidase	IDUA	P35475	CVD II	100.00%
Tyrosine-protein kinase receptor Tie-1	TIE1	P35590	C-Met	100.00%
Glutaredoxin-1	GLRX	P35754	Met	100.00%
Vascular endothelial growth factor receptor 3	VEGFR-3	P35916	Onc II	86.98%
Vascular endothelial growth factor receptor 2	VEGFR-2	P35968	Onc II	100.00%
Chitinase-3-like protein 1	CHI3L1	P36222	CVD III	100.00%
Lymphotoxin-beta receptor	LTBR	P36941	CVD III	100.00%
Serine/threonine-protein kinase receptor R3	SKR3	P37023	Neuro	99.90%
TGF-beta receptor type-2	TGFR-2	P37173	Onc II	100.00%
Collagen alpha-1(XVIII) chain	COL18A1	P39060	C-Met	100.00%
Matrix metalloproteinase-12	MMP-12	P39900	CVD II	95.25%
Macrophage-capping protein	CAPG	P40121	OD	98.76%
Alpha-taxilin	TXL	P40222	Onc II	99.38%
B-cell antigen receptor complex-associated protein beta chain	CD79B	P40259	Met	99.17%
OX-2 membrane glycoprotein	CD200	P41217	Neuro	100.00%
Protein phosphatase inhibitor 2	PPP1R2	P41236	Met	100.00%
Neuroblastoma suppressor of tumorigenicity 1	NBL1	P41271	Neuro	100.00%
Folate receptor gamma	FR-gamma	P41439	Onc II	100.00%
Caspase-3	CASP-3	P42574	CVD III	99.90%
Dipeptidyl aminopeptidase-like protein 6	DPP6	P42658	OD	99.90%
Leukemia inhibitory factor receptor	LIF-R	P42702	Inf	100.00%
Lysosomal Pro-X carboxypeptidase	PRCP	P42785	C-Met	98.66%
C-X-C motif chemokine 5	CXCL5	P42830	Inf	100.00%
Cathepsin O	CTSO	P43234	Met	100.00%
Tumor necrosis factor receptor superfamily member 4	TNFRSF4	P43489	Onc II	99.79%
Neurogenic locus notch homolog protein 1	NOTCH1	P46531	C-Met	100.00%
Lymphotactin	XCL1	P47992	CVD II	99.90%
Fas antigen ligand	FasL	P48023	Onc II	100.00%
Carboxypeptidase A2	CPA2	P48052	Neuro	99.90%
Tissue factor pathway inhibitor 2	TFPI-2	P48307	Onc II	100.00%
Protein NOV homolog	NOV	P48745	Dev	100.00%
CD97 antigen	CD97	P48960	Dev	100.00%
Cartilage oligomeric matrix protein	COMP	P49747	C-Met	100.00%
Placenta growth factor	PGF	P49763	CVD II, OD	100.00%

Fms-related tyrosine kinase 3 ligand	Flt3L	P49771	Inf	100.00%
Serpin B8	SERPINB8	P50452	Met	100.00%
Methionine aminopeptidase 2	MetAP 2	P50579	Onc II	98.04%
TNF-related apoptosis-inducing ligand	TRAIL	P50591	Inf, Onc II	100%; 99,9%
Basal cell adhesion molecule	BCAM	P50895	Dev	100.00%
Eotaxin	CCL11	P51671	Inf, IR	100%; 99,79%
Amyloid-like protein 1	APLP1	P51693	Met	100.00%
Prolargin	PRELP	P51888	CVD II	100.00%
Dual specificity mitogen-activated protein kinase kinase 6	MAP2K6	P52564	Cell-Reg	96.90%
Ephrin-A4	EF4	P52798	Neuro	99.90%
Stanniocalcin-1	STC1	P52823	IR	100.00%
Thimet oligopeptidase	THOP1	P52888	Met	100.00%
Dipeptidyl peptidase 1	CTSC	P53634	Neuro	99.90%
Ephrin type-B receptor 4	EPHB4	P54760	CVD III	100.00%
Phospholipid transfer protein	PLTP	P55058	C-Met	100.00%
Mesencephalic astrocyte-derived neurotrophic factor	MANF	P55145	Neuro	99.90%
Cadherin-6	CDH6	P55285	Neuro	100.00%
C-C motif chemokine 23	CCL23	P55773	Inf	100.00%
C-C motif chemokine 18	CCL18	P55774	C-Met	100.00%
Glycoprotein Xg	XG	P55808	Dev	99.79%
GDNF family receptor alpha-1	GFR-alpha-1	P56159	Neuro	99.90%
Galectin-4	Gal-4	P56470	CVD III	90.29%
Junctional adhesion molecule B	JAM-B	P57087	Neuro	100.00%
Protein FAM3B	FAM3B	P58499	IR	100.00%
Thymosin beta-10	TMSB10	P63313	Dev	100.00%
Coxsackievirus and adenovirus receptor	CXADR	P78310	IR	100.00%
Tyrosine-protein phosphatase non-receptor type substrate 1	SHPS-1	P78324	CVD III	99.90%
Disintegrin and metalloproteinase domain-containing protein 8	ADAM8	P78325	Onc II	99.28%
Glypican-5	GCP5	P78333	Neuro	100.00%
Lectin-like oxidized LDL receptor 1	LOX-1	P78380	CVD II	100.00%
Butyrophilin subfamily 3 member A2	BTN3A2	P78410	IR	100.00%
Fractalkine	CX3CL1	P78423	Inf	100.00%
Interleukin-13 receptor subunit alpha-1	IL13RA1	P78552	Dev	94.11%
Monocyte chemotactic protein 2	MCP-2	P80075	Inf	100.00%
C-X-C motif chemokine 6	CXCL6	P80162	Inf	100.00%
Nucleobindin-2	NUCB2	P80303	OD	99.79%
Protein delta homolog 1	DLK-1	P80370	CVD III	100.00%
Perlecan	PLC	P98160	CVD III	100.00%
CD83 antigen	CD83	Q01151	IR	100.00%
ST2 protein	ST2	Q01638	CVD III	93.70%
Inactive tyrosine-protein kinase transmembrane receptor ROR1	ROR1	Q01973	Met	100.00%
N-acylethanolamine-hydrolyzing acid amidase	NAAA	Q02083	Neuro	99.69%
Contactin-2	CNTN2	Q02246	OD	100.00%
Desmocollin-2	DSC2	Q02487	Dev	100.00%
Angiopoietin-1 receptor	TIE2	Q02763	CVD II	100.00%
Peptidyl-prolyl cis-trans isomerase FKBP4	FKBP4	Q02790	Met	100.00%
Transforming growth factor beta receptor type 3	TGFBR3	Q03167	C-Met	100.00%
Trefoil factor 2	TFF2	Q03403	Met	99.90%
Urokinase plasminogen activator surface receptor	U-PAR	Q03405	CVD III	100.00%
Parathyroid hormone/parathyroid hormone-related peptide receptor	PTH1R	Q03431	IR	86.04%
Lactoylglutathione lyase	GLO1	Q04760	CVD II	100.00%
Sialomucin core protein 24	CD164	Q04900	Met	100.00%
Tyrosine-protein kinase receptor TYRO3	TYRO3	Q06418	Met	100.00%
Peroxiredoxin-1	PRDX1	Q06830	IR	100.00%
Tumor necrosis factor receptor superfamily member 9	TNFRSF9	Q07011	Inf	100.00%
Cytoskeleton-associated protein 4	CKAP4	Q07065	IR	100.00%
Early activation antigen CD69	CD69	Q07108	Dev	100.00%
C-X-C motif chemokine 9	CXCL9	Q07325	Inf	100.00%
Trefoil factor 3	TFF3	Q07654	CVD III	100.00%
Interleukin-10 receptor subunit beta	IL-10RB	Q08334	Inf	100.00%
Epithelial discoidin domain-containing receptor 1	DDR1	Q08345	Neuro	100.00%
Lactadherin	MFGE8	Q08431	Dev	100.00%
Testican-1	SPOCK1	Q08629	Neuro	100.00%
CMRF35-like molecule 6	CLM-6	Q08708	Neuro	99.90%
Polypeptide N-acetylgalactosaminyltransferase 2	GALNT2	Q10471	Cell-Reg	100.00%
CMP-N-acetylneuraminate-beta-galactosamide-alpha-2,3-sialyltransferase 1	ST3GAL1	Q11201	OD	99.69%
EGF-containing fibulin-like extracellular matrix protein 1	EFEMP1	Q12805	C-Met	100.00%
Contactin-1	CNTN1	Q12860	CVD III	100.00%
Tyrosine-protein kinase Mer	MERTK	Q12866	CVD II	95.76%
BMP and activin membrane-bound inhibitor homolog	BAMBI	Q13145	OD	99.79%
Pappalysin-1	PAPPA	Q13219	CVD II	100.00%
Chitotriosidase-1	CHIT1	Q13231	CVD III	96.38%
Natural killer cells antigen CD94	KLRD1	Q13241	IR	92.55%
Semaphorin-3F	SEMA3F	Q13275	Met	100.00%
Inactive tyrosine-protein kinase 7	PTK7	Q13308	OD	94.52%
Receptor-type tyrosine-protein phosphatase S	PTPRS	Q13332	C-Met	100.00%
Microfibrillar-associated protein 5	MFAP5	Q13361	C-Met	99.79%
Mesothelin	MSLN	Q13421	Onc II	99.90%
Interleukin-18 receptor 1	IL-18R1	Q13478	Inf	100.00%
Eukaryotic translation initiation factor 4E-binding protein 1	4E-BP1	Q13541	Inf	100.00%
CD166 antigen	ALCAM	Q13740	CVD III	100.00%
Bleomycin hydrolase	BLM hydrolase	Q13867	CVD III	100.00%
Pro-interleukin-16	IL16	Q14005	CVD II	94.32%
Lysosome membrane protein 2	SCARB2	Q14108	Neuro	99.90%

Nidogen-2	NID2	Q14112	Dev	100.00%
Interleukin-18	IL-18	Q14116	Inf, CVD II	100%; 99,59%
Dystroglycan	DAG1	Q14118	Dev	100.00%
Scavenger receptor class F member 1	SCARF1	Q14162	Dev	100.00%
P-selectin glycoprotein ligand 1	PSGL-1	Q14242	CVD II	100.00%
Growth arrest-specific protein 6	GAS6	Q14393	C-Met	100.00%
WAP four-disulfide core domain protein 2	WFDC2	Q14508	Onc II	100.00%
SPARC-like protein 1	SPARCL1	Q14515	C-Met	100.00%
LDLR chaperone MESD	MESDC2	Q14696	Dev	100.00%
Latent-transforming growth factor beta-binding protein 2	LTBP2	Q14767	C-Met	100.00%
Procollagen C-endopeptidase enhancer 1	PCOLCE	Q15113	C-Met	100.00%
Nodal modulator 1	NOMO1	Q15155	Met	100.00%
Serum paraoxonase/arylesterase 2	PON2	Q15165	OD	94.94%
Paraoxonase	PON3	Q15166	CVD III	92.87%
Receptor tyrosine-protein kinase erbB-4	ErbB4/HER4	Q15303	Onc II	100.00%
Angiopoietin-1	ANG-1	Q15389	CVD II	100.00%
Ficolin-2	FCN2	Q15485	C-Met	92.36%
Transforming growth factor-beta-induced protein ig-h3	TGFBI	Q15582	C-Met	100.00%
Cystatin-M	CST6	Q15828	Dev	100.00%
Clusterin-like protein 1	CLUL1	Q15846	Met	100.00%
Insulin-like growth factor-binding protein 7	IGFBP-7	Q16270	CVD III	100.00%
NT-3 growth factor receptor	NTRK3	Q16288	Neuro	99.90%
Laminin subunit alpha-4	LAMA4	Q16363	Dev	100.00%
BDNF/NT-3 growth factors receptor	NTRK2	Q16620	Neuro	100.00%
C-C motif chemokine 14	CCL14	Q16627	C-Met	100.00%
Prostasin	PRSS8	Q16651	CVD II	100.00%
Myelin-oligodendrocyte glycoprotein	MOG	Q16653	Cell-Reg	100.00%
C-C motif chemokine 15	CCL15	Q16663	CVD III	100.00%
Melanoma-derived growth regulatory protein	MIA	Q16674	Onc II	100.00%
Kynureninase	KYNU	Q16719	Neuro	100.00%
Glutaminyl-peptide cyclotransferase	QPCT	Q16769	C-Met	100.00%
Kynurenine--oxoglutarate transaminase 1	KYAT1	Q16773	Met	100.00%
Carbonic anhydrase IX	CAIX	Q16790	Onc II	100.00%
Membrane primary amine oxidase	AOC3	Q16853	C-Met	99.59%
MHC class I polypeptide-related sequence A/B	MIC-A/B	Q29983,Q29980	Onc II	99.59%
R-spondin-1	RSPO1	Q2MKA7	Neuro	98.76%
Brorin	VWC2	Q2TAL6	Neuro	99.90%
Protogenin	PRTG	Q2VWP7	Neuro	99.90%
Cell adhesion molecule-related/down-regulated by oncogenes	CDON	Q4KMG0	Dev	99.79%
Collectin-12	COLEC12	Q5KU26	Dev	100.00%
Platelet endothelial aggregation receptor 1	PEAR1	Q5VY43	Dev	100.00%
Meteorin-like protein	METRNL	Q641Q3	Met	100.00%
Vasorin	VASN	Q6EMK4	C-Met	100.00%
Leukocyte-associated immunoglobulin-like receptor 1	LAIR1	Q6GTX8	Dev	100.00%
Leukocyte-associated immunoglobulin-like receptor 2	LAIR-2	Q6ISS4	Neuro	99.48%
RGM domain family member B	RGMB	Q6NW40	Neuro	100.00%
Layilin	LAYN	Q6UX15	Neuro	100.00%
VEGF-co regulated chemokine 1	CXL17	Q6UXB2	Onc II	99.28%
Seizure 6-like protein 2	SEZ6L2	Q6UXD5	Cell-Reg	100.00%
CMRF35-like molecule 9	CD300LG	Q6UXG3	Dev	97.31%
Cysteine-rich with EGF-like domain protein 2	CRELD2	Q6UXH1	Dev	100.00%
Inactive serine protease PAMR1	PAMR1	Q6UXH9	Dev	100.00%
Leucine-rich repeat neuronal protein 1	LRRN1	Q6UXK5	Cell-Reg	100.00%
Chordin-like protein 2	CHRDL2	Q6WN34	Met	95.56%
CD109 antigen	CD109	Q6YHK3	Dev	100.00%
Scavenger receptor class A member 5	SCARA5	Q6ZMJ2	Neuro	99.90%
Coiled-coil domain-containing protein 80	CCDC80	Q76M96	Met	100.00%
Protein FAM19A5	FAM19A5	Q7Z5A7	Cell-Reg	100.00%
Allergin-1	MILR1	Q7Z6M3	IR	100.00%
Amphoterin-induced protein 2	AMIGO2	Q86SJ2	Cell-Reg	100.00%
Polypeptide N-acetylgalactosaminyltransferase 10	GALNT10	Q86SR1	OD	99.79%
C-type lectin domain family 14 member A	CLEC14A	Q86T13	Dev	100.00%
Scavenger receptor cysteine-rich type 1 protein M130	CD163	Q86VB7	CVD III	100.00%
Low-density lipoprotein receptor-related protein 11	LRP11	Q86VZ4	Met	100.00%
Interferon lambda receptor 1	IFNLR1	Q8IU57	IR	99.69%
Plexin domain-containing protein 1	PLXDC1	Q8IUK5	OD	99.79%
Contactin-4	CNTN4	Q8IWV2	Dev	100.00%
SIR2-like protein 2	SIRT2	Q8IXJ6	Inf	97.73%
Osteoclast-associated immunoglobulin-like receptor	hOSCAR	Q8IYS5	CVD II	100.00%
Cell adhesion molecule 3	CADM3	Q8N126	Neuro	100.00%
Leukocyte immunoglobulin-like receptor subfamily B member 2	LILRB2	Q8N423	C-Met	99.59%
Inactive dipeptidyl peptidase 10	DPP10	Q8N608	IR	100.00%
CD177 antigen	CD177	Q8N6Q3	Dev	95.66%
Draxin	DRAXIN	Q8NBI3	Neuro	100.00%
Sulfatase-modifying factor 2	SUMF2	Q8NBJ7	Met	100.00%
Proprotein convertase subtilisin/kexin type 9	PCSK9	Q8NBP7	CVD III	99.79%
Thioredoxin domain-containing protein 5	TXNDC5	Q8NBS9	Met	100.00%
Interleukin-27	IL-27	Q8NEV9,Q14213	CVD II	100.00%
MAM domain-containing glycosylphosphatidylinositol anchor protein 1	MDGA1	Q8NFP4	Neuro	100.00%
Leukocyte immunoglobulin-like receptor subfamily B member 4	LILRB4	Q8NHJ6	IR	100.00%
Leukocyte immunoglobulin-like receptor subfamily B member 1	LILRB1	Q8NHL6	C-Met	100.00%
Multiple coagulation factor deficiency protein 2	MCFD2	Q8NI22	Met	100.00%
Interleukin-17D	IL-17D	Q8TAD2	CVD II	100.00%
CD99 antigen-like protein 2	CD99L2	Q8TCZ2	Dev	100.00%

Cell surface glycoprotein CD200 receptor 1	CD200R1	Q8TD46	Neuro	98.04%
Hepatitis A virus cellular receptor 2	HAVCR2	Q8TDQ0	Dev	100.00%
CMRF35-like molecule 1	CLM-1	Q8TDQ1	Neuro	99.90%
A disintegrin and metalloproteinase with thrombospondin motifs 15	ADAM-TS 15	Q8TE58	Onc II	100.00%
WAP, Kazal, immunoglobulin, Kunitz and NTR domain-containing protein 2	WFIKKN2	Q8TEU8	Dev	100.00%
Soluble calcium-activated nucleotidase 1	CANT1	Q8WVQ1	Met	100.00%
Insulin-like growth factor-binding protein-like 1	IGFBPL1	Q8WX77	Met	100.00%
Protein FAM3C	FAM3C	Q92520	Met	100.00%
C-C motif chemokine 17	CCL17	Q92583	CVD II	99.90%
Nectin-2	NECTIN2	Q92692	Met	100.00%
Tenascin-R	TN-R	Q92752	Neuro	100.00%
Secreted frizzled-related protein 3	sFRP-3	Q92765	Neuro	100.00%
Neuronal cell adhesion molecule	Nr-CAM	Q92823	Neuro	100.00%
Kallikrein-6	KLK6	Q92876	CVD III	100.00%
Tumor necrosis factor receptor superfamily member 14	TNFRSF14	Q92956	CVD III	100.00%
Tumor necrosis factor receptor superfamily member 19L	RELT	Q969Z4	Dev	100.00%
Endothelial cell-selective adhesion molecule	ESAM	Q96AP7	Dev	100.00%
Repulsive guidance molecule A	RGMA	Q96B86	Neuro	100.00%
Kidney Injury Molecule	KIM1	Q96D42	CVD II, OD	100%; 97,12%
Interleukin-17 receptor A	IL-17RA	Q96F46	CVD III	100.00%
Scavenger receptor class F member 2	SCARF2	Q96GP6	Neuro	99.90%
Brevican core protein	BCAN	Q96GW7	Neuro	100.00%
T-cell immunoglobulin and mucin domain-containing protein 4	TIMD4	Q96H15	C-Met	91.01%
Kazal-type serine protease inhibitor domain-containing protein 1	KAZALD1	Q96I82	Cell-Reg	99.59%
Leucine-rich repeats and immunoglobulin-like domains protein 1	LRIG1	Q96JA1	Met	100.00%
Beta-Ala-His dipeptidase	CNDP1	Q96KN2	C-Met	100.00%
Fc receptor-like protein 1	FCRL1	Q96LA6	Met	92.77%
Sialic acid-binding Ig-like lectin 10	SIGLEC10	Q96LC7	Cell-Reg	100.00%
Nectin-4	PVRL4	Q96NY8	Onc II	99.90%
WAP, Kazal, immunoglobulin, Kunitz and NTR domain-containing protein 1	WFIKKN1	Q96NZ8	Neuro	92.25%
Discoidin, CUB and LCCL domain-containing protein 2	DCBLD2	Q96PD2	IR	93.38%
Secretoglobin family 3A member 2	SCGB3A2	Q96PL1	CVD III	86.98%
VPS10 domain-containing receptor SorCS2	SORCS2	Q96PQ0	Cell-Reg	100.00%
Probable carboxypeptidase X1	CPXM1	Q96SM3	Cell-Reg	100.00%
Proheparin-binding EGF-like growth factor	HB-EGF	Q99075	CVD II	100.00%
Protein deglycase DJ-1	PARK7	Q99497	Dev	100.00%
Sortilin	SORT1	Q99523	CVD II	100.00%
Legumain	LGMN	Q99538	Dev	100.00%
Oncostatin-M-specific receptor subunit beta	OSMR	Q99650	C-Met	99.90%
Mothers against decapentaplegic homolog 5	MAD homolog 5	Q99717	Onc II	100.00%
Metalloproteinase inhibitor 4	TIMP4	Q99727	CVD III	100.00%
C-C motif chemokine 19	CCL19	Q99731	Inf	100.00%
Chymotrypsin C	CTRC	Q99895	CVD II	98.86%
Retinoic acid receptor responder protein 2	RARRES2	Q99969	CVD III	100.00%
Myocilin	MYOC	Q99972	Dev	100.00%
Osteomodulin	OMD	Q99983	Dev	100.00%
Growth/differentiation factor 15	GDF-15	Q99988	CVD III	100.00%
Sclerostin	SOST	Q9BQB4	Met	100.00%
Serine protease 27	PRSS27	Q9BQR3	CVD II	100.00%
Calsyntenin-3	CLSTN3	Q9BQT9	Cell-Reg	100.00%
Spondin-2	SPON2	Q9BUD6	CVD II	100.00%
Brother of CDO	Protein BOC	Q9BWV1	CVD II, Cell-Reg	100%; 100%
Complement C1q tumor necrosis factor-related protein 1	C1QTNF1	Q9BXJ1	C-Met	100.00%
C-type lectin domain family 7 member A	CLEC7A	Q9BXN2	IR	99.28%
Complement factor H-related protein 5	CFHR5	Q9BXR6	C-Met	100.00%
R-spondin-3	RSPO3	Q9BXY4	Onc II	100.00%
Angiopoietin-related protein 4	ANGPTL4	Q9BY76	Dev	100.00%
Seizure 6-like protein	SEZ6L	Q9BYH1	Onc II	100.00%
Regenerating islet-derived protein 4	REG4	Q9BYZ8	Met	100.00%
NKG2D ligand 2	N2DL-2	Q9BZM5	Neuro	99.90%
Reticulon-4 receptor	RTN4R	Q9BZR6	Met	100.00%
Natural killer cell receptor 2B4	CD244	Q9BZW8	Inf	99.48%
Sialoadhesin	SIGLEC1	Q9BZZ2	Neuro	99.90%
Semaphorin-4C	SEMA4C	Q9C0C4	Cell-Reg	99.90%
Tubulointerstitial nephritis antigen-like	TIGL1	Q9GZM7	Met	100.00%
SLIT and NTRK-like protein 2	SLITRK2	Q9H156	Cell-Reg	100.00%
Multiple epidermal growth factor-like domains protein 9	MEGF9	Q9H1U4	C-Met	100.00%
C-X-C motif chemokine 16	CXCL16	Q9H2A7	CVD III	100.00%
Transmembrane protease serine 5	TMPRSS5	Q9H3S3	Neuro	100.00%
SPARC-related modular calcium-binding protein 2	SMOC2	Q9H3U7	Neuro	100.00%
Calsyntenin-2	CLSTN2	Q9H4D0	Met	100.00%
CUB domain-containing protein 1	CDCP1	Q9H5V8	Inf	100.00%
SLIT and NTRK-like protein 6	SLITRK6	Q9H5Y7	Cell-Reg	91.83%
CXADR-like membrane protein	CLMP	Q9H6B4	Met	100.00%
Tumor necrosis factor receptor superfamily member 27	EDA2R	Q9HAV5	Neuro	99.90%
Interleukin-1 receptor-like 2	IL1RL2	Q9HB29	CVD II	99.79%
T-lymphocyte surface antigen Ly-9	LY9	Q9HBG7	Onc II	89.88%
Spondin-1	SPON1	Q9HCB6	CVD III	100.00%
Roundabout homolog 2	ROBO2	Q9HCK4	Neuro	99.90%
Plexin-A4	PLX4	Q9HCM2	IR	100.00%
Resistin	RETN	Q9HD89	CVD III	100.00%
CD209 antigen	CD209	Q9NNX6	Dev	100.00%
Tumor necrosis factor receptor superfamily member 12A	TNFRSF12A	Q9NP84	Neuro	99.90%
Triggering receptor expressed on myeloid cells 1	TREM1	Q9NP99	IR	98.76%

Lysophosphatidic acid phosphatase type 6	ACP6	Q9NPH0	Met	100.00%
Complement component C1q receptor	CD93	Q9NPY3	CVD III	100.00%
Endothelial cell-specific molecule 1	ESM-1	Q9NQ30	Onc II	100.00%
Cartilage acidic protein 1	CRTAC1	Q9NQ79	C-Met	100.00%
Fructose-2,6-bisphosphatase TIGAR (TIGAR)	TIGAR	Q9NQ88	OD	88.43%
Neural proliferation differentiation and control protein 1	NPDC1	Q9NQX5	Met	100.00%
Neutral ceramidase	N-CDase	Q9NR71	Neuro	99.69%
C-C motif chemokine 28	CCL28	Q9NRJ3	Inf	97.00%
Interleukin-17 receptor B	IL17RB	Q9NRM6	Cell-Reg	100.00%
Tumor necrosis factor receptor superfamily member 19	TNFRSF19	Q9NS68	Onc II	100.00%
Fibroblast growth factor 21	FGF-21	Q9NSA1	Cell-Reg	86.76%
Phosphoprotein associated with glycosphingolipid-enriched microdomains 1	PAG1	Q9NWQ8	Met	100.00%
C-type lectin domain family 5 member A	CLEC5A	Q9NY25	Met	100.00%
Podocalyxin-like protein 2	PODXL2	Q9NZ53	Cell-Reg	100.00%
Programmed cell death 1 ligand 1	PD-L1	Q9NZQ7	Inf	100.00%
Cysteine-rich motor neuron 1 protein	CRIM1	Q9NZV1	Dev	100.00%
Kallikrein-14	hK14	Q9P0G3	Onc II	96.80%
Disintegrin and metalloproteinase domain-containing protein 22	ADAM 22	Q9P0K1	Neuro	100.00%
C-type lectin domain family 1 member B	CLEC1B	Q9P126	Neuro	97.52%
Dickkopf-related protein 3	DKK3	Q9UBP4	Dev	100.00%
Cathepsin Z	CTSZ	Q9UBR2	CVD III	100.00%
Dickkopf-related protein 4	Dkk-4	Q9UBT3	Neuro	99.90%
Cathepsin F	CTSF	Q9UBX1	Dev	100.00%
Kallikrein-11	hK11	Q9UBX7	Onc II	100.00%
Macrophage receptor MARCO	MARCO	Q9UEW3	CVD II	100.00%
Contactin-associated protein-like 2	CNTNAP2	Q9UHC6	IR	100.00%
Epidermal growth factor-like protein 7	EGFL7	Q9UHF1	OD	99.59%
Dipeptidyl peptidase 2	DPP7	Q9UHL4	Met	99.48%
Adhesion G protein-coupled receptor E2	ADGRE2	Q9UHX3	Met	100.00%
SLAM family member 5	CD84	Q9UIB8	CVD II	99.38%
Paired immunoglobulin-like type 2 receptor beta	PILRB	Q9UKJ0	Met	100.00%
Paired immunoglobulin-like type 2 receptor alpha	PILRA	Q9UKJ1	Dev	100.00%
ADP-sugar pyrophosphatase	NUDT5	Q9UKK9	Dev	97.93%
Kallikrein-13	KLK13	Q9UKR3	Onc II	100.00%
Plexin-B3	PLXNB3	Q9ULL4	Neuro	100.00%
Carbonic anhydrase 14	CA14	Q9ULX7	OD	99.79%
Neurogenic locus notch homolog protein 3	Notch 3	Q9UM47	CVD III	100.00%
Tumor necrosis factor receptor superfamily member EDAR	EDAR	Q9UNE0	IR	99.07%
Syntaxin-8	STX8	Q9UNK0	OD	99.79%
C-type lectin domain family 11 member A	CLEC11A	Q9Y240	Dev	100.00%
Tumor necrosis factor ligand superfamily member 13B	TNFSF13B	Q9Y275	CVD III	100.00%
V-set and immunoglobulin domain-containing protein 4	VSIG4	Q9Y279	Dev	99.90%
Sialic acid-binding Ig-like lectin 7	SIGLEC7	Q9Y286	Met	100.00%
Sialic acid-binding Ig-like lectin 9	Siglec-9	Q9Y336	Neuro	100.00%
CD2-associated protein	CD2AP	Q9Y5K6	Met	92.67%
Ectonucleoside triphosphate diphosphohydrolase 2	ENTPD2	Q9Y5L3	OD	99.48%
Wnt inhibitory factor 1	WIF-1	Q9Y5W5	Onc II	100.00%
Lymphatic vessel endothelial hyaluronic acid receptor 1	LYVE1	Q9Y5Y7	C-Met	100.00%
Junctional adhesion molecule A	JAM-A	Q9Y624	CVD III	100.00%
Adhesion G-protein coupled receptor G1	ADGRG1	Q9Y653	OD	99.38%
Roundabout homolog 1	ROBO1	Q9Y6N7	Dev	100.00%
Tumor necrosis factor receptor superfamily member 11A	TNFRSF11A	Q9Y6Q6	CVD II	100.00%
Delta and Notch-like epidermal growth factor-related receptor	DNER	Q8NFT8	Inf	100.00%
Transmembrane glycoprotein NMB	GPNMB	Q14956	Onc II	100.00%
Galactoside 3(4)-L-fucosyltransferase,Alpha-(1,3)-fucosyltransferase 3/5	(FUT3/5)	Q11128,P21217	Dev	95.86%
Fibroblast growth factor 5 (FGF-5)	FGF-5	P12034	Inf	100.00%

Table shows those proteins that passed the qusliy control criteria and were detected in more than 85% of the samples. Panel Key: CVD II = Cardiovascular disease II; CVD III =Cardiovasculr disease III; C-Met = Cardiometabolic; Cell-Reg = Cell Regulation; Dev = Development; Inf = Inflammation; IR = Immune Response; I/O = Immuno-Oncology; Met = Metabolism; Neuro = Neurology; OD = Organ Damage; Onc II = Oncology II
