# Supplementary material for: CSF proteome profiling reveals biomarkers to discriminate dementia with Lewy bodies from Alzheimer´s disease
Source: Nat Commun. 2023 Sep 13;14:5635. doi: 10.1038/s41467-023-41122-y (PMC10499811; doi:10.1038/s41467-023-41122-y)
Supplement: Supplementary file 3 — Description of Additional Supplementary Files [file 41467_2023_41122_MOESM3_ESM.pdf]

Description of Additional Supplementary Files

File Name: Supplementary Data 1

Description: Supplementary data set shows the effect (beta coefficient), p- and q- values of each of the CSF protein abundance that differed between DLB (n=109) and CON (n=190) or AD (n=235) or AD and CON after two-sided nested F-test analysis and FDR post-hoc correction. Significant proteins (q <0.05) are highlighted in bold.
