# Supplementary material for: CSF proteome profiling reveals biomarkers to discriminate dementia with Lewy bodies from Alzheimer´s disease
Source: Nat Commun. 2023 Sep 13;14:5635. doi: 10.1038/s41467-023-41122-y (PMC10499811; doi:10.1038/s41467-023-41122-y)
Supplement: Supplementary file 5 — Reporting Summary [file 41467_2023_41122_MOESM5_ESM.pdf]

nature portfolio

Corresponding author(s): Marta del Campo

Last updated by author(s): 03/08/2023

Reporting Summary

Nature Portfolio wishes to improve the reproducibility of the work that we publish. This form provides structure for consistency and transparency in reporting. For further information on Nature Portfolio policies, see our Editorial Policies and the Editorial Policy Checklist.

Statistics

For all statistical analyses, confirm that the following items are present in the figure legend, table legend, main text, or Methods section.

n/a	Confirmed	
☐	☒	The exact sample size (*n*) for each experimental group/condition, given as a discrete number and unit of measurement
☐	☒	A statement on whether measurements were taken from distinct samples or whether the same sample was measured repeatedly
☐	☒	The statistical test(s) used AND whether they are one- or two-sided<br>*Only common tests should be described solely by name; describe more complex techniques in the Methods section.*
☐	☒	A description of all covariates tested
☐	☒	A description of any assumptions or corrections, such as tests of normality and adjustment for multiple comparisons
☐	☒	A full description of the statistical parameters including central tendency (e.g. means) or other basic estimates (e.g. regression coefficient) AND variation (e.g. standard deviation) or associated estimates of uncertainty (e.g. confidence intervals)
☐	☒	For null hypothesis testing, the test statistic (e.g. *F*, *t*, *r*) with confidence intervals, effect sizes, degrees of freedom and *P* value noted<br>*Give P values as exact values whenever suitable.*
☒	☐	For Bayesian analysis, information on the choice of priors and Markov chain Monte Carlo settings
☒	☐	For hierarchical and complex designs, identification of the appropriate level for tests and full reporting of outcomes
☐	☒	Estimates of effect sizes (e.g. Cohen's *d*, Pearson's *r*), indicating how they were calculated

Our web collection on statistics for biologists contains articles on many of the points above.

Software and code

Policy information about availability of computer code

Data collection	Olink® NPX Manger software
Data analysis	Statistical analyses were performed using SPSS (version 25; SPSS/IBM, Chicago, IL), R (version 3.5.3) and metascape (metascape.org). Classification models were built using glmnet R package.

For manuscripts utilizing custom algorithms or software that are central to the research but not yet described in published literature, software must be made available to editors and reviewers. We strongly encourage code deposition in a community repository (e.g. GitHub). See the Nature Portfolio guidelines for submitting code & software for further information.

Data

Policy information about availability of data

All manuscripts must include a data availability statement. This statement should provide the following information, where applicable:

- Accession codes, unique identifiers, or web links for publicly available datasets
- A description of any restrictions on data availability
- For clinical datasets or third party data, please ensure that the statement adheres to our policy

The DLB data generated in this study have been deposited in the synapse database under accession code https://www.synapse.org/PRIDE_DLB. The PD data used in the preparation of this article were obtained from the Parkinson's Progression Markers Initiative (PPMI) database (www.ppmi-info.org/access-data-specimens/

download-data). For up-to-date information on the study, visit www.ppmi-info.org.

Human research participants

Policy information about studies involving human research participants and Sex and Gender in Research.

Reporting on sex and gender	Analysis of covariance was performed when an association between classical AD CSF biomarker and age and/or sex were detected. For each individual protein feature of the CSF proteome PEA data we assessed if its addition to a base model containing age and gender contributed to model fit. Classification modeling was performed by penalized generalized linear modeling (GLM) with an elastic net penalty (a linear combination of lasso and ridge penalties) in the discovery CSF cohort using the glmnet package and including age and sex as covariates.<br>The demographic characteristics of all the cohorts used in this study are included in Table 1.
Population characteristics	The discovery cohort of this study included CSF samples from 109 patients with DLB(18/101 (F/M), Avg Age 69), 235 with AD(96/139 (F/M), Avg Age 66), and 190 cognitively unimpaired controls (70/120 (F/M), Avg Age 58). Three additional independent CSF cohorts were used for validation. The clinical validation cohort 1 included CSF samples from 54 patients with DLB (24/69 (F/M), Avg Age 69), 55 with AD (23/35 (F/M), Avg Age 66) and 55 controls (22/33 (F/M), Avg Age 58). The clinical validation cohort 2 included CSF samples from 55 patients with DLB (22/55 (F/M), Avg Age 76), 55 patients with AD (34/21 (F/M), Avg Age 72), and 55 controls (32/23 (F/M), Avg Age 62). The autopsy confirmed cohort included CSF samples from 17 cases with DLB (3/14 (F/M), Avg Age 76) and 30 with AD (15/15 (F/M), Avg Age 71). An additional 29 cognitively unimpaired controls (13/16 (F/M), Avg Age 63) from the same center were included in this cohort but these were not autopsy confirmed.
Recruitment	Most of the samples were selected from the Amsterdam Dementia Cohort (ADC) and DEvELOP. To enrich for DLB dementia cases, additional CSF samples from the Center for Neurodegenerative Disease Research at the University of Pennsylvania were included. The three additional independent CSF cohorts used for validation from ADC (clinical validation 1, total n=164; all groups), Sant Pau Initiative on Neurodegeneration (SPIN) cohort (clinical validation cohort 2; total n=165) and BIODEM and the neurobiobank of the Institute Born-Bunge (IIB) / UAntwerp (autopsy confirmed cohort; total n=76). All participants of every cohort underwent standard neurological and cognitive assessments and diagnosis was assigned according to international consensus criteria for DLB and AD. The neuropathological validation cohort included cases with a definite diagnosis according to international neuropathological examination guidelines for DLB and AD. Mini-Mental State Examination (MMSE) was used as a measure of global cognition. Motor parkinsonism was assessed using section III of the Unified Parkinson's Disease Rating Scale (UPDRS). Core and supportive clinical features34 were recorded locally in each memory unit26,27,29. Evaluation of REM Sleep Behavior Disorder (RBD) includes an interview with a sleep specialist, a full nocturnal video-polysomnography, the Mayo Sleep questionnaire (cutoff ≥1) or caregivers reporting that a patient would 'act out' their dreams and moves extensively during their sleep as previously described. Supportive neuropsychiatry symptoms (e.g., depression, delusions, apathy, anxiety) were compiled according to the Neuropsychiatry Inventory and summarized in one single score (NPI-total). Levels of CSF Aβ42, tTau and pTau(181) ('AD CSF biomarkers') were used to support AD diagnoses. The control group included individuals with subjective cognitive decline, in whom objective cognitive and laboratory investigations were normal (i.e., criteria for MCI, dementia, or any other neurological or psychiatric disorder not fulfilled) with additionally negative AD CSF biomarkers. Participants provided written informed consent at the time of recruitment. Patients were recruited from specialized memory units associated to academic research centers and are extensively and throughly characterized, and thus may not reflect the entirely population.
Ethics oversight	The studies were approved by the Institutional Ethical Review Boards of each center (VUmc: AD CSF biobank METC number 00-211; University of Pennsilvania: language and cognitive impairment in parkinson's disease and parkinson's disease with dementia or dementia with lewy bodies IRB069801; SPIN cohort: COLLECTION 16/2013)

Note that full information on the approval of the study protocol must also be provided in the manuscript.

Field-specific reporting

Please select the one below that is the best fit for your research. If you are not sure, read the appropriate sections before making your selection.

☒ Life sciences ☐ Behavioural & social sciences ☐ Ecological, evolutionary & environmental sciences

For a reference copy of the document with all sections, see nature.com/documents/nr-reporting-summary-flat.pdf

Life sciences study design

All studies must disclose on these points even when the disclosure is negative.

Sample size	No sample size calculation was performed. Sample size was chosen based on resources and CSF samples that were readily available in our biobanks.
Data exclusions	Proteins with values below the lower limit of detection (LOD) in more than 85% of the samples were excluded from the analysis.
Replication	The cross-validation methods employed support the reproducibility of the CSF panels identified. Three independent cohorts were included to

Replication	validated the panels identified. The protein effect sizes obtained with these custom assays in the three validation cohorts correlated well with those obtained in the discovery cohort (r coefficients ranging between 0.70 and 0.99), and the high discriminative values were mostly validated (AUCs > 0.80), supporting the relevance and robustness of the findings.
Randomization	Samples were randomized across plates containing appropriate inter-plate quality controls from manufacturer and measured in two different rounds. Each round included 16 bridging samples covering different clinical groups which were used for reference sample normalization to control for potential batch effects.
Blinding	CSF measurements were conducted by manufacturer who has no access to any clinical data or group information.

Reporting for specific materials, systems and methods

We require information from authors about some types of materials, experimental systems and methods used in many studies. Here, indicate whether each material, system or method listed is relevant to your study. If you are not sure if a list item applies to your research, read the appropriate section before selecting a response.

Materials & experimental systems

n/a	Involved in the study
☐	☒ Antibodies
☒	☐ Eukaryotic cell lines
☒	☐ Palaeontology and archaeology
☒	☐ Animals and other organisms
☒	☐ Clinical data
☒	☐ Dual use research of concern

Methods

n/a	Involved in the study
☒	☐ ChIP-seq
☒	☐ Flow cytometry
☒	☐ MRI-based neuroimaging

Antibodies

Antibodies used	Multiplex antibody-based protein panels based on the proximity extension assay (PEA) (Cardiometabolic, Cardiovascular II and III, cell regulation, development, immune response, inflammation, metabolism, neurology, oncology II and organ damage; Olink proteomics, Uppsala, Sweden (www.olink.com). CSF Aβ(1-42), total and phosphorylated Tau were analyzed locally as part of the routine diagnostic procedure using commercially available kits (VUmc and UAntwerp: ELISA INNOTEST Aβ(1-42), hTAUAg, phospho-Tau(181P, Fujirebio, Ghent, Belgium) or VUmc: Aβ(1-42), t-TAUAg, phospho-Tau181 Elecsys biomarker assays (Roche Diagnostics GmbH); Penn: Luminex xMAP INNO-BIA AlzBio3; Luminex Corp, Austin, TX; SPIN: Lumipulse G600, Fujirebio).
Validation	All characteristics and validation data for each Olink assay are available at the manufacturer´s webpage (www.olink.com). CSF Aβ(1-42), total and phosphorylated Tau 181 have been extensively validated and are routinely used for research and clinical purposes.
